# Supplementary material for: Quantification of Microsphere Drug Release by Fluorescence Imaging with the FRET System
Source: Pharmaceutics. 2024 Jul 31;16(8):1019. doi: 10.3390/pharmaceutics16081019 (PMC11360167; doi:10.3390/pharmaceutics16081019)
Supplement: Supplementary file 1 [file pharmaceutics-16-01019-s001.zip › pharmaceutics-3053888-supplementary.pdf]

Supplementary Information

For

Quantification of Microsphere Drug Release by Fluorescence Imaging with the FRET System

Yuying Chen [1], Huangjie Lu [1], Qingwei He [1], Jie Yang [1], Hong Lu [1], Jiongming Han [2], Ying Zhu [1] and Ping Hu [1,*]

[1] College of Pharmacy, Jinan University, Guangzhou 511436, China; cyy19981209@stu2021.jnu.edu.cn (Y.C.); tyxx70126@stu2021.jnu.edu.cn (H.L.); heqingwei524@163.com (Q.H.); msjieyang@163.com (J.Y.); luhong0910@foxmail.com (H.L.); 17670960705@163.com (Y.Z.)

[2] International School, Jinan University, Guangzhou 511436, China; 18203931589@163.com

* Correspondence: inzahu@hotmail.com or pinghu@jnu.edu.cn; Tel.: +86-18581483142

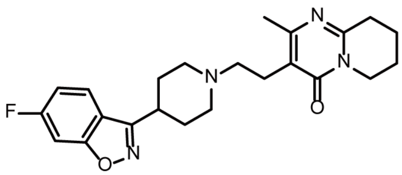

Risperidone

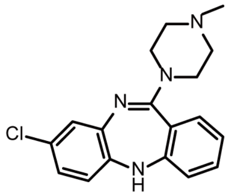

Clozapine

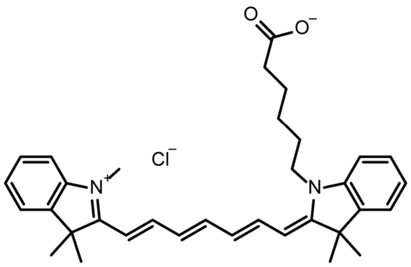

Cy5

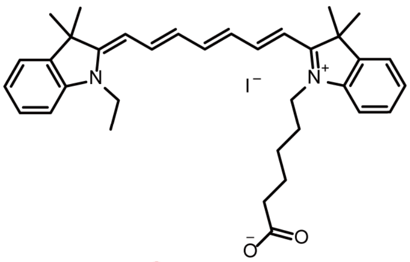

Cy7

Figure S1: Chemical structures of Risperidone, Clozapine and Cyanine dyes.

Table S1: Molecular weights and Log P values of Risperidone, Clozapine and Cyanine dyes

chemical compound	molecular weight	Log P
Risperidone	410.48	2.02
Clozapine	326.82	3.71
Cyanine 5	519.12	3.31
Cyanine 7	650.63	2.97
